# Robustness and Uncertainties of the “Temperature and Greenness” Model for Estimating Terrestrial Gross Primary Production

**DOI:** 10.1038/srep44046

**Published:** 2017-03-08

**Authors:** Jiaqi Dong, Longhui Li, Hao Shi, Xi Chen, Geping Luo, Qiang Yu

**Affiliations:** 1 State Key Laboratory of Desert and Oasis Ecology, Xinjiang Institute of Ecology and Geography, Chinese Academy of Sciences, Urumqi 830011, China; 2 Graduate School, University of Chinese Academy of Sciences, Beijing 100100, China; 3 State Key Laboratory of Soil Erosion and Dryland Farming on the Loess Plateau, Northwest A & F University, Yangling, China

## Abstract

Terrestrial gross primary production (GPP) plays a vital role in offsetting anthropogenic CO_2_ emission and regulating global carbon cycle. Various remote sensing approaches for estimating GPP have attracted considerable scientific attentions, yet their robustness and uncertainties remain unclear. Here we evaluate the performance of the “temperature and greenness” (TG) model, a representative remote sensing model in estimating GPP, using the global FLUXNET GPP based on parameter sensitive analysis and optimization strategies. The results show that the minimum (x_n_) and optimum (x_o_) temperatures for photosynthesis are sensitive parameters but maximum temperature (x_m_) not. Optimized x_n_ and x_o_ differ largely from their defaults for more than half of 12 plant functional types (PFTs). Parameter optimization significantly improves the TG’s performance in forest ecosystems where temperature or solar radiation has significant contribution to GPP. For water-limited ecosystems where GPP are strongly dependent of EVI and EVI are sensitive to precipitation, parameter optimization has limited effects. These results imply that the TG model, and most likely for other kind of GPP models using same methodology, can’t be significantly improved for all PFTs through parameter optimization only, and other key climatic variables should be incorporated into the model for better predicting terrestrial ecosystem GPP.

Terrestrial gross primary production (GPP) is the major driver of global carbon cycle and it plays an important role in regulating the concentration of CO_2_ of the atmosphere by partly offsetting anthropogenic CO_2_ emissions^1^. However, direct measurements of GPP are not available, because no observational techniques are ready to quantify this process at the right spatial scale^2^.

Quantification of GPP at ecosystem level is mostly inferred from the measurements of net ecosystem production (NEP) between terrestrial ecosystems and the atmosphere using the eddy covariance (EC) equipment^3^. More than 950 site-years EC data have been archived in the international network of FLUXNET^4^ during the past three decades, which make the estimation of GPP possible at site level. However, estimate of GPP at a larger (for example regional, national or global) scale was difficult^5^. To retrieve GPP estimates at larger scales, site level of EC-inferred GPP have to be scaled up to spatial domains based on empirically statistical methods^5^,^6^, such as artificial neural networks^7^ or ensemble model trees^5^, or semi-empirical models including light use efficiency^8^ or water-use efficiency^9^ approaches. A common disadvantage of these approaches is the strong dependency on environmental (vegetation, soil or meteorological) variables^6^.

Another commonly used approach to quantify GPP based on land surface models (LSMs) or ecosystem models at different scales^10^. These models have explicit modules to simulate carbon cycle processes by describing plant physiological behaviour in relation to soil and atmospheric processes^11^. Models can be implemented alone (offline)^12^,^13^,^14^ or coupled with climate model (online, also termed Earth System Models)^15^,^16^,^17^. The significant advantage of these carbon models relies in the continuousness in both space and time, so that they have been often used to detect the trend and inter-annual variability of GPP in long term period and at larger scales^18^,^19^. However, LSMs require multiple driving data including meteorological variables, vegetation and soil maps, which are highly spatial heterogeneities and uncertainties.

Alternatively, GPP at larger scale can be estimated by the approach using satellite measurements of vegetation parameters that are directly related to plant photosynthesis (i.e., GPP)^20^. These parameters mainly include photosynthetically active radiation (PAR), normalized difference vegetation index (NDVI) or enhanced vegetation index (EVI), and leaf area index (LAI). Integrating one or more of these photosynthesis-related remote sensing measurements into an empirical or semi-empirical model was used to predict GPP. Amongst existed models, a classical type is termed light use efficiency (LUE) model, which describes GPP as a multiple product of PAR, fraction of PAR absorbed by the vegetation (fPAR), potential LUE (LUE_max_) and the environmental constraints on LUE_max_^21^,^22^,^23^,^24^,^25^,^26^. LUE-type models also require ancillary environmental variables such air temperature, vapor pressure deficit, soil moisture or canopy water content to constrain LUE_max_^26^. In LUE-type models, fPAR was generally calculated as a linear function of NDVI^27^. However, NDVI was found to be easily saturated at moderate to high vegetation coverage^28^,^29^,^30^. Development of EVI, in certain extent, reduced the severity of early saturation^31^. EVI was found to perform better than NDVI in predicting GPP in high dense vegetation, such as evergreen and deciduous forests^32^,^33^ and cropland as well^34^. Based on strong correspondence of GPP on EVI over one or two week intervals^35^,^36^, a cluster of EVI-based models was developed to predict GPP^32^,^33^,^35^,^36^,^37^. Amongst the EVI-based models, temperature and greenness (TG) model received much attention due to its simplicity which uses purely remote sensed land surface temperature (LST) and EVI, free of any ancillary meteorological data, as drivers^37^. In the TG model, LST was considered to be representative of climatic variables and EVI include information of photosynthetic potential^37^. In addition, the TG model only consists of three temperature-related parameters (see Methods), which represent the minimum (x_n_), optimum (x_o_) and maximum (x_m_) LST values on photosynthesis, respectively. The TG model has been widely evaluated against with site level of derived GPP from EC^38^,^39^ and inter-compared with other LUE models^39^, it showed significant advantages in limited ecosystem types. However, the robustness of such a simply model in estimating GPP across a wide spectrum of ecosystems and associated uncertainties from model parameters have not yet been investigated.

Therefore, the first objective of this research is to conduct a comprehensive evaluation of the TG model for predicting GPP using the EC-derived GPP. The second objective is to deploy the potential of parameter optimization method in improving the performance of the TG model. Finally, this paper aims at investigating the robustness and uncertainties of the TG model among different biomes through introducing other climatic factors (e.g., precipitation and solar radiation) into a linearly multiple regression model. Such evaluation and investigation provided a common methodology to any other LUE-type models for predicting GPP, although the TG model was adopted as a case in this research.

## Results

### Parameter sensitivities of the TG model

**Figure 1:**
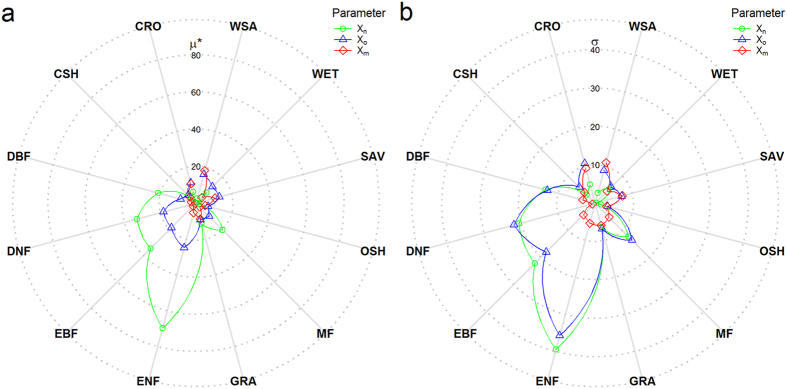
Mean elementary effects (μ*) (**a**) and standard deviation of elementary effects (σ) (**b**) derived from the Morris screening analysis for twelve PFTs. Each axis of the radar plot corresponds to the values of μ* or σ and different colors of lines with symbols represent three parameters of the TG model. A larger μ*, the more important the parameter in the TG model for a specific PFT. Legends for PFTs were defined in Methods.

The TG model consists of three parameters, x_n_, x_o_ and x_m_, which represent minimum, optimum and maximum temperature for plant photosynthesis^37^. Their values were set as 0, 30, and 50 °C, respectively, in the TG model^37^. The three parameters are referred to LST, rather than air temperature, which is more commonly used to drive productivity models^37^. The values of the three temperature-related parameters may differ largely across PFTs, so we set wide ranges of the three parameters (Table 1) to investigate their sensitivities using Morris method^40^. From the Morris screening method, two indices μ* and σ measure the influence of each parameter and its degree of involvement in non-linearities and interactions with other parameters, respectively. None of σ value was significantly larger than 2 μ* for all parameters and all PFTs, indicating that limited interactions and/or non-linearities existed (Fig. 1). Then we ranked the relative degree of sensitivities of three parameters based on their μ* values for each PFT and a larger μ* was considered as a more influential parameter (Fig. 1a). It is clearly that mostly influential parameters differ across PFTs. Parameter x_n_ and x_o_ were identified as mostly influential parameters of the TG model for all forests (including DBF, DNF, EBF, ENF and MF) and GRA, while x_o_ was selected as the relatively influential parameters for other remaining PFTs (including CSH, CRO, WSA, WET, SAV and OSH). However, x_m_ was not identified as the mostly influential parameter for any PFT (Fig. 1 and Fig. S1).

### Optimization of parameters of the TG model

Theoretically, only those parameters selected as mostly influential by the Morris screening analysis were applied to MCMC for parameter optimization. Because there were only three parameters in the TG model and applying all three parameters to MCMC will not exert great burdens on computation, we included all three parameters (x_n,_ x_o_ and x_m_) during MCMC optimization.

**Figure 2:**
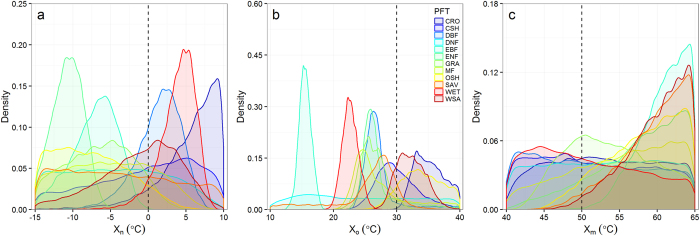
Density of the posterior parameters for three parameters (**a**) for x_n,_ (**b**) for x_o_ and (**c**) for x_m_ of the TG model. Lines with different colors represent different PFTs. Three vertically dashed lines represent the default values for parameter x_n_, x_o_ and x_m_ of the TG model, respectively. Legends for PFTs were defined in Methods.

To guarantee the validity of the optimized parameters from MCMC, convergence of the posterior parameters must be assured. A mostly direct way to diagnose the convergence is visually plotting the density of the posterior parameter. More like a Gaussian distribution of the posterior parameter, more possible the convergence can be assured. As shown in Fig. 2a, parameter x_n_ was identified as convergent for ENF, EBF, DBF and WET. Parameter x_o_ was considered as convergent for all PFTs except CRO, DNF and OSH (Fig. 2b). However, x_m_ was identified as unconvergent during the range of 40–65 °C for all PFTs (Fig. 2c) due to their uniform or gamma distributions. The convergence of the posterior parameters optimized by MCMC agreed with the results diagnosed by running mean of the posterior parameters (Fig. S2). Overall, posterior distribution of parameter x_o_ showed convergence in nine of twelve PFTs (excluding CRO, DNF and OSH), and that of parameter x_n_ showed convergence in four of twelve PFTs (including ENF, EBF, DBF and WET) but posterior distribution of x_m_ from MCMC did not show convergence for any PFT. It is worth mentioning that the parameters identified as convergent from MCMC (Fig. 2) were consistent with those parameters identified as mostly influential by the Morris screening analysis (Fig. 1a), which also indicated the advantage of applying all three parameters to MCMC.

**Figure 3:**
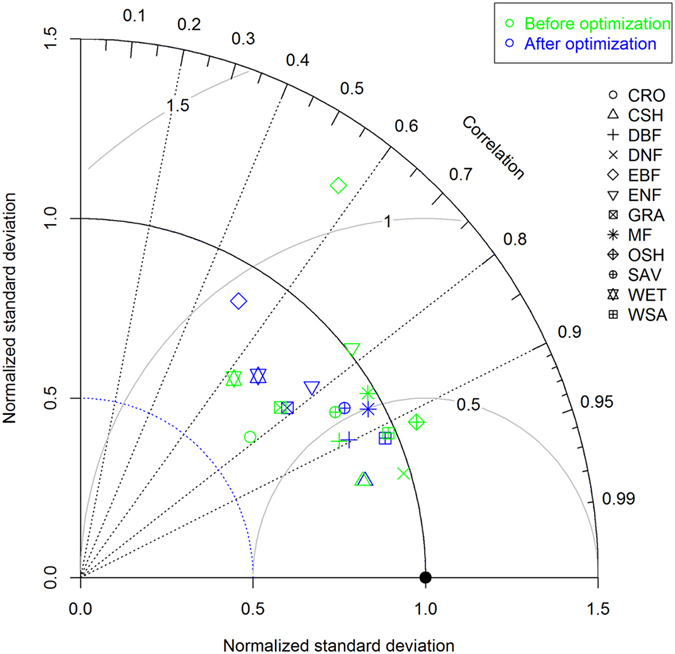
Effects of MCMC optimization on performance of the TG model in twelve PFTs. Twelve points with different shapes represent PFTs and green and blue color points represent the performance of the TG model before and after MCMC optimization. The black solid circle on the x-axis represents the reference (observation) with normalized standard deviation and correlation coefficient (Correlation) with 1.0 and 1.0, respectively. The solid gray arch lines labeled with digits represent the normalized room mean square difference. Legends for PFTs were defined in Methods.

The optimized parameter value for those PFTs which were sensitive to x_n_ were significantly different from the default value of x_n_ (0 °C), and had wide ranges from −10 °C in ENF to 5 °C in WET (Fig. 2a). For parameter x_o_, the optimized value by MCMC differed largely across PFTs (Fig. 2b). In EBF, the optimized value for x_o_ was 15 °C, half of the default value (30 °C) of the TG model. In WET, the optimized x_o_ was 22.5 °C. In DBF, ENF, GRA and MF, the optimized x_o_ by MCMC were about 25 °C. While, in the remaining three PFTs (CSH, SAV and WSA) which were sensitive to x_o_, the optimized values of x_o_ ranged from 27.5 to 32.5 °C, and were close to the default x_o_ (30 °C) of the TG model. Wide ranges of the optimized x_o_ for LST by MCMC provided good support for previous report that optimum air temperature for canopy carbon flux varied from 7.5 to 30 °C and were highly correlated with mean summer temperature^41^. For parameter x_m_, no optimized value can be derived by the MCMC method during the range of LST between 40 and 65 °C (Fig. 3c), which was in good agreement with the analysis from the Morris screening method (Fig. 1).

### Effects of MCMC optimization on the TG model

Figure 3 shows the performance of the TG model before and after MCMC optimization. The default TG model (before optimization) performed large difference in different PFT. Correlation coefficient (R) between the observed GPP and the simulated GPP by the default TG model ranged from 0.55 in EBF to 0.96 in DNF, but were larger than 0.7 in ten of twelve PFTs (except EBF and WET), which indicated that the default TG model was able to explain more than 60% of variance of the observed GPP at monthly scale. After applying MCMC to the TG model, correlation coefficient (R) between data and model did not significantly increase in all twelve PFTs, but both normalized standard deviation (STD) and root mean squared difference (RMSD) decreased in EBF and ENF. STD decreased from 1.32 to 0.90, and RMSD decreased from 1.12 to 0.94 for EBF. For ENF, STD decreased from 1.01 to 0.86, and RMSD decreased from 0.67 to 0.62. Obviously, the effects of MCMC optimization on performance of the TG model were caused by the difference in the values of parameters x_n_ and/or x_o_ of the model (Fig. 2a and b). Optimized TG model by MCMC did not cause the increase in R between data and model, but caused the decrease in normalized STD and RMSD in EBF and ENF, which were also attributed by the nature that the maximum likelihood estimation was used as a cost function in MCMC.

### Relative Contributions of EVI and Climatic Variables on GPP

**Figure 4:**
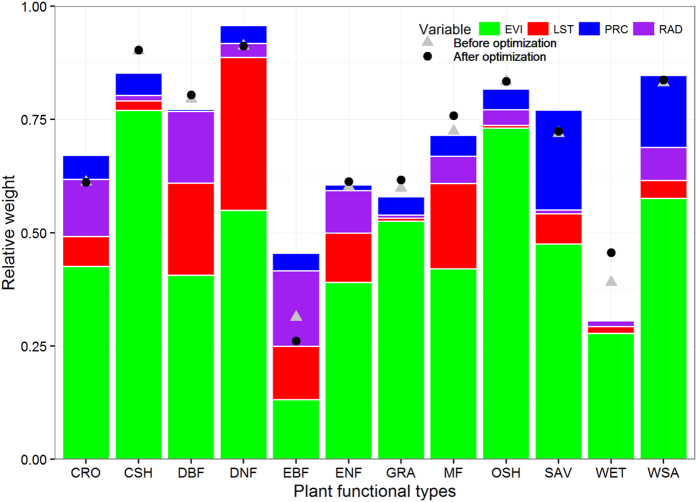
Relative contributions of EVI (green), land surface temperature (LST, red), precipitation (PRC, blue) and radiation (RAD, purple) on GPP in twelve plant functional types (PFTs). Gray triangle and black circle represent correlation coefficient (R) between the data and the model simulated by the default and optimized TG model with MCMC method, respectively. Legends for PFTs were defined in Methods.

It is clear that EVI had dominated effects on GPP in all PFTs except EBF in which RAD was found to have comparable effects on GPP (Fig. 4). Individual effect of EVI was able to contribute 13% and 28% of GPP variation in EBF and WET, respectively. In the remaining ten PFTs, EVI could contribute 39% (ENF) to 77% (CSH) of GPP variations. When introducing LST as a predictor to estimate GPP in MLR, LST showed its significant effects on GPP in forest PFTs (including DBF, DNF, EBF, ENF and MF) but insignificant in non-forest PFTs. Similarly, RAD was found to have significant effects to estimate GPP in forest PFTs (excluding DNF with limited measurements) which are mostly energy limited ecosystems and moderate effects on CRO and WSA as well. In contrast, PRC was found to have significant effects on GPP in WSA, SAV, OSH, CSH and CRO, in which GPP were mostly possible to be limited by water^42^.

Given considering the four key variables (EVI, LST, PRC and RAD) together as predictors for estimating GPP using MLR, we found that the MLR using the four variables had comparable capability to estimate GPP as the TG model in ten out of twelve PFTs except EBF and WET. In EBF, MLR using four variables had worse ability to estimate GPP but better in WET.

## Discussions

Although the TG model was reported to have high accuracy of predicting GPP across a wide range of ecosystem types, previous evaluations were mostly focused on limited ecosystem types or few sites of data for an ecosystem type^37^,^39^. This study made utilize of a global FLUXNET GPP data to demonstrate that the TG model performed well (R^2^ > 0.7) for 7 of 12 PFTs (CSH, DFB, DNF, MF, OSH, SAV, and WSA), and moderately (0.5 < R^2^ < 0.7) for 3 PFTs (CRO, GRA and ENF), but poorly (R^2^ < 0.5) for EBF and WET. So it is necessary to understand why the TG model performed differently across PFTs.

Overall, the TG model predicted GPP well compared with the flux tower GPP in WSA and SAV where vegetation are sparse, which obviously conflicted with previous understanding^37^. It was deemed that solar elevation angel strongly affected the EVI values when vegetation is sparse^37^ which are cases for WSA and SAV. Our analysis showed that EVI was still well correlated with GPP in SAV and WSA, indicating that vegetation fraction coverage may be not an important source of errors. More importantly, EVI was proven to be very sensitive to PRC in SAV and WSA^43^, so LST was found to be have less contribution than PRC on GPP (Fig. 4). This is also evidenced in CSH, GRA and OSH. However, in DBF, EBF and ENF, RAD was found to have considerable contributions on GPP, indicating that GPP of forest ecosystems may be limited by RAD^44^. In WET, GPP was principally influenced by RAD^45^ and non-climatic variables such as leaf area index and water table depth^45^. As the number of GPP data for DNF was relatively limited, RAD did not show significant contribution on GPP.

The performance of a model can be determined by the model structure (behaviour), model parameters and input^10^. In this study, we mainly focused on the effects of model parameters on the performance of the TG model by optimizing the parameter based on MCMC. In terms of parameter values in the TG model, optimized values for both x_o_ and x_n_ differed largely from their default ones for more than half of 12 PFTs. However, parameter optimization using MCMC significantly improved the model’s performance for EBF and ENF only, where temperature and solar radiation are critically important for GPP. For other ecosystem types, such as DBF and WET, MCMC did not significantly improve the performance identified by a Taylor diagram, although the optimized parameters (x_o_ and x_n_) values were largely different from their default. This suggests that the performance of the TG model can’t be improved by optimizing its parameters only, which also reflected that the TG model has large potential to improve in model structure and process. For example, solar radiation should be integrated into the model for forested ecosystems, and precipitation should be considered for water-limited ecosystems (WSA, SAV, and GRA). However, in some water-limited ecosystems, EVI was found to be very sensitive to precipitation^43^. Consequently, the TG model performed very well for these water-limited ecosystems (Fig. 2). However, we should note that in some PFTs, MCMC did not really improve the model performance but still induce a large discrepancy in the values of parameters. This suggests that the optimized parameter values may not correspond to true underlying values. Another reason is that the TG model was jointly determined by all parameters, rather than any individual one. Therefore, the optimized value for a single parameter by MCMC should not be over-interpreted.

Similar to previously studies using vegetation index to predict GPP^46^,^47^, LUE-type models showed robustness for some ecosystems and weakness as well for other ecosystems or when drought events presented. The essential causes were that the algorithms describing the environmental regulations, particularly water stress on GPP built in those models are largely different. By comparing the TG model (with or without parameter optimization) with a linearly multiple regression model, other climatic variables, such as radiation or precipitation, have significant effects on GPP in some ecosystems. In the TG model, LST was considered to be representative of climatic conditions (air temperature, vapor pressure deficit, or radiation). However, this prerequisite for the TG model does not hold for all ecosystems, evidenced by largely different “relative contribution” of each climatic variable to GPP (Fig. 4). This implies that the TG model, and most likely for other kind of GPP models using same methodology, should incorporate other key climatic variables for further improving the accuracy in simulating terrestrial ecosystem GPP.

## Methods

### Global GPP dataset

The analyses performed in this study are based on monthly GPP data from 155 flux towers, consisting of a total of 624 site-year datasets and representing a world wide spectrum of biomes and climate regions. These data covered 12 ecosystem types including cropland (CRO), closed shrubland (CSH), deciduous broadleaf forest (DBF), deciduous needleleaf forest (DNF), evergreen broadleaf forest (EBF), evergreen needleleaf forest (ENF), grassland (GRA), mixed forest (MF), open shrubland (OSH), savanna (SAV), wetland (WET), and wood savanna (WSA). Geophysical location of the flux tower sites are shown in the Fig. S3, and the number of each ecosystem type is listed in the Table S1. The majority of data were obtained from global FLUXNET (La Thule level 2 or level 4 products)^48^, and the rest of data were obtained directly from the site researchers. GPP data were inferred from net ecosystem production (NEP) observed by eddy covariance. Only site-years with small gaps (i.e., individual gaps in NEP of less than 5% of the entire annual record) were selected except in certain ecosystems of the boreal region where only growing season data were available. Missing values of NEP (and GPP) were gap-filled and partitioned by a publicly available marginal distribution sample (MDS) online tool^3^.

### Satellite data

MODIS monthly enhanced vegetation index (EVI) (MOD13A3.005) and land surface temperature (LST) (MOD11C3.005) products for February 2000 to 2013 were obtained from the USGS repository (http://e4ftl01.cr.usgs.gov/MOLT/). Both EVI and LST dataset were produced globally over land at 1-km resolution. Due to the difficulty of precisely co-locating the pixels that directly correspond to the footprint of an EC flux site, a central 3 × 3 km window surrounding the flux tower was used to extract mean EVI and LST time series^49^. To reduce noise and uncertainties in the MODIS EVI and LST time series at each site, the singular spectrum analysis (SSA) was employed. In the monthly EVI and LST time series, a window length of 37 (i.e. 37 months) and 6 leading components were found to best captured the periodicity and simultaneously reduced random noises during reconstruction. To further minimize the contamination, the snow/ice flag in MOD13A3 quality assurance field was first used to remove the snow-covered EVI values; and then EVI values were further screened for effects of cold temperature (LST < −2.0 °C) using the MODIS LST^50^.

### Temperature and greenness (TG) model

The temperature and greenness (TG) model simulates GPP as a proportionally linear function of the product of scaled EVI (EVI_scaled_) and LST (LST_scaled_)^37^, i.e.,


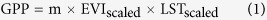


where m is a scalar with unit of gC m^−2^ day^−1^.

The EVI scalar is simply defined as:


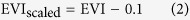


where 0.1 was the EVI value when GPP drops to zero.

Another scalar LST_scaled_ was defined as:


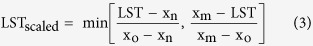


where min represent the minimum value of the two items in the square brackets, x_n_, x_o_ and x_m_ are three parameters, and represent minimum, optimum and maximum temperature for plant photosynthesis^37^. Their default values were set as 0, 30, and 50 °C, respectively^37^. In the TG model, LST represent climatic conditions in some extent, and EVI is used as a measure of vegetation greenness.

### Ranking of parameter sensitivities

Although there are only three parameters in the TG model, it is still necessary to understand the sensitivity of the model to each parameter. Here we use the Morris method^40^,^51^ to screen out the most important parameters. The Morris screening is based on a one-at-a-time (OAT) approach, in which only one parameter is varied between two runs, allowing for calculating a local partial derivative of the output variable with respect to the input parameter. This impact of input parameter on the output is called elementary effect. The Morris method calculates the elementary effects of each parameter in several locations of the parameter space several times, so it is generally considered to be a “global” screening method. The mean (μ*) and standard deviation (σ) of all elementary effects for each parameter can be used to be representative of the behavior of this parameter in its entire range of variation. The two indices μ* and σ measure the influence of the parameter on output variable and its degree of involvement in non-linearities and/or interactions with other parameters, respectively.

In this study, the R package “sensitivity”^52^ was used to implement the Morris method for the TG model across 12 ecosystem types.

### Bayesian approach for parameter optimization

Parameter optimization was performed within a Bayesian framework, which treats the unknown model parameters x as random variables with the posterior probability density function (pdf) *p(x*|*y*) given by


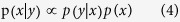


where *p(x*) denotes the prior distribution of *x* and *p(y*|*x*) signifies the likelihood function of *x*. Bayesian inference was performed using Markov Chain Monte Carlo (MCMC)^53^. For the application of Bayes theorem, an efficient algorithm called the Metropolis-Hastings algorithm^54^,^55^ was used to the posterior distribution *p(x*|*y*). The prior probability density function was first specified by providing a set of limiting intervals for the parameters. The likelihood function was then constructed assuming that errors in the observed data followed Gaussian distributions. In total, 10^5^ iterations of probability distribution for each PFT were carried out to best satisfy convergence.

### Statistical analysis

The Taylor diagram^56^ was used to describe the effects of parameter optimization on the performance of the TG model. The performance of the TG model was displayed in a single diagram, featured by correlation coefficient (R), standard error (STD), and root mean square difference (RMSD). The higher the R and the smaller the STD and RMSD, the better the agreement between model and data is. When comparing two simulations with different parameter values, i.e. the default TG model or the TG model after MCMC optimization, the longer the distance between the two simulation points, the greater effect of optimization on the model’s performance. To make the performance of the TG model across ecosystems, the Taylor diagram was shown in a normalized way, so “observations” are displayed in the point (R = 1, STD = 1, RMSD = 0).

To investigate why the TG model performed differently amongst PFTs, relative weight analysis (RWA) was used to quantify the relative contributions of EVI and three key climatic variables, LST, precipitation (PRC) and radiation (RAD) on GPP, supplementary to multiple linear regression (MLR) analysis. The function “rlw” in the R package “yhat” was used to implement RWA, and the R package “plotrix” was used to plot Taylor diagram.

## Additional Information

**How to cite this article:** Dong, J. *et al*. Robustness and uncertainties of the “temperature and greenness” model for estimating terrestrial gross primary production. *Sci. Rep.* **7**, 44046; doi: 10.1038/srep44046 (2017).

**Publisher's note:** Springer Nature remains neutral with regard to jurisdictional claims in published maps and institutional affiliations.

## Supplementary Material

Supplementary Information

## Figures and Tables

**Table 1:** Default, ranges and optimized values for the three parameters of the TG model for 12 PFTs.

Parameter	Default	Ranges	Optimized											
			CRO	CSH	DBF	DNF	EBF	ENF	GRA	MF	OSH	SAV	WET	WSA
x_n_	0	−15–10	0	0	2.5	0	−6	−10	0	0	0	0	5	0
x_o_	30	10–40	30	29	27	30	15	27	27	24	30	28	22	31
x_m_	50	40–65	50	50	50	50	50	50	50	50	50	50	50	50

x_n_, x_o_ and x_m_ represent the minimum, optimal and maximum land surface temperature for photosynthesis. All variables are united in °C. Legends for PFTs were defined in Methods.
