# Supplementary material for: Robustness and Uncertainties of the “Temperature and Greenness” Model for Estimating Terrestrial Gross Primary Production
Source: Sci Rep. 2017 Mar 8;7:44046. doi: 10.1038/srep44046 (PMC5341022; doi:10.1038/srep44046)
Supplement: Supplementary Information [file srep44046-s1.pdf]

Supplementary Information

Robustness and uncertainties of the "temperature and greenness" model for estimating terrestrial gross primary production

Jiaqi Dong[1, 2], Longhui Li[1, *], Hao Shi[3], Xi Chen[1], Geping Luo[1], Qiang Yu[3]

[1] State Key Laboratory of Desert and Oasis Ecology, Xinjiang Institute of Ecology and Geography, Chinese Academy of Sciences, Urumqi 830011, China

[2] Graduate School, University of Chinese Academy of Sciences, Beijing 100100, China

[3] State Key Laboratory of Soil Erosion and Dryland Farming on the Loess Plateau, Northwest A & F University, Yangling, China

This supplementary information includes:

Figure S1-S3.

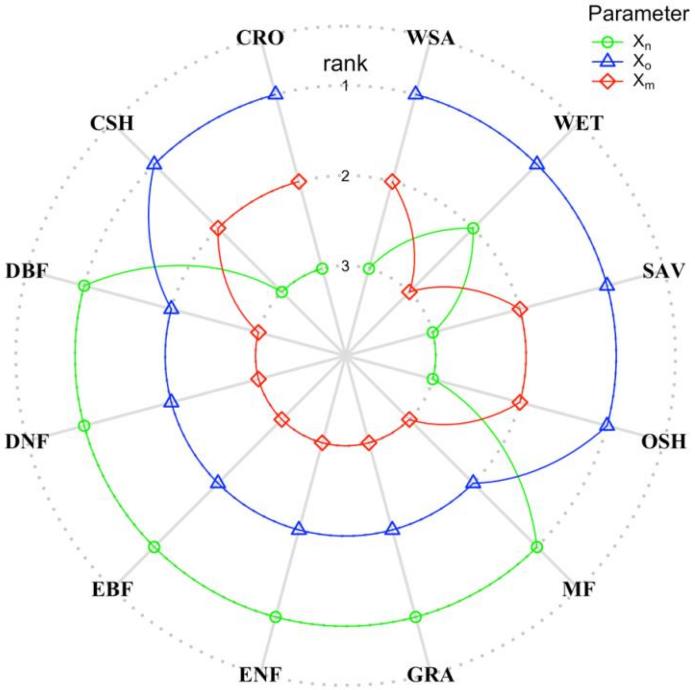

Figure S1. Parameters rankings derived from the Morris screening analysis for the TG model. Each axis of the radar plot corresponds to the rank of a parameter, the lower the rank, the more important the parameter.

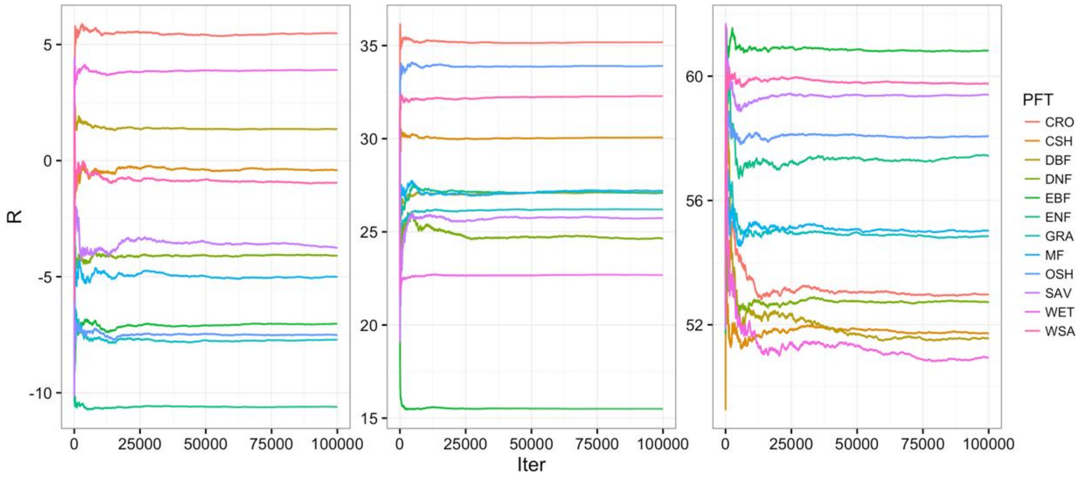

Figure S2. The track plots for accepted x_n, x_o and x_m values in the Bayesian MCMC optimization. 100,000 iterations were conducted for each parameter within 12 biomes to approach convergence.

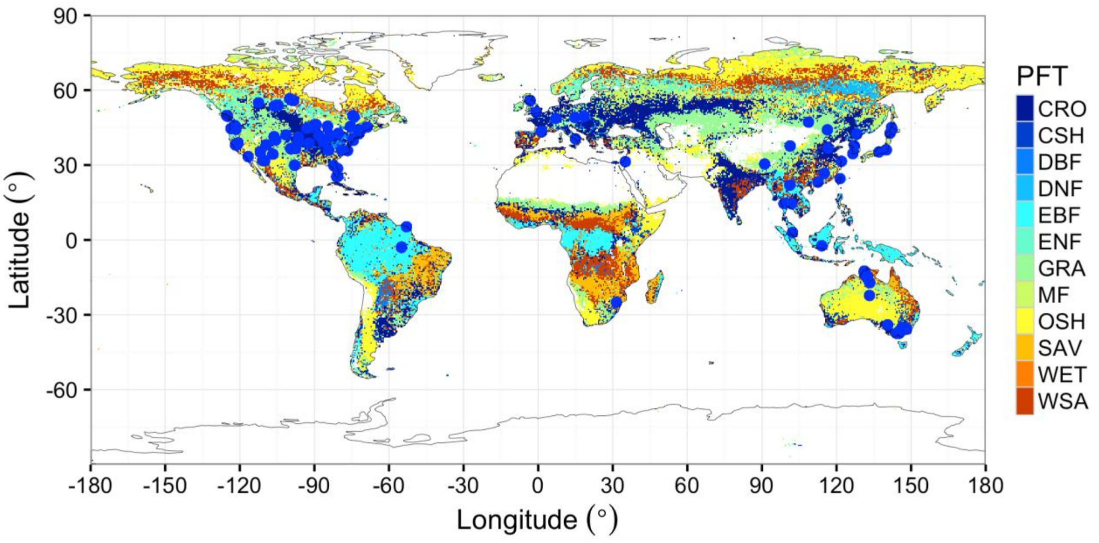

Figure S3. Geographical distribution of flux towers (blue filled circle) overlaid onto the 2001 MODIS IGBP land cover map at a 0.5º × 0.5º resolution. Map was drawn using R version 3.2.4 (http://www.R-project.org/).
